# What Matters 2 Adults: a study protocol to develop a new preference-based wellbeing measure with Aboriginal and Torres Strait Islander adults (WM2Adults)

**DOI:** 10.1186/s12889-020-09821-z

**Published:** 2020-11-17

**Authors:** Kirsten Howard, Kate Anderson, Joan Cunningham, Alan Cass, Julie Ratcliffe, Lisa J. Whop, Michelle Dickson, Rosalie Viney, Brendan Mulhern, Allison Tong, Gail Garvey

**Affiliations:** 1 School of Public Health, Faculty of Medicine and Health, University of Sydney, Sydney, Australia; 2 Menzies School of Health Research, Charles Darwin University, Darwin, Australia; 3 Health and Social Care Economics Group, Caring Futures Institute, Flinders University, Adelaide, Australia; 4 National Centre for Epidemiology and Population Health, Australian National University, Canberra, Australia; 5 Centre for Health Economics Research and Evaluation, UTS Business School, University of Technology Sydney, Sydney, Australia

**Keywords:** Wellbeing, Aboriginal and Torres Strait islander peoples, Preferences, Values

## Abstract

**Background:**

Understandings of health and wellbeing are culturally bound. Many Aboriginal and Torres Strait Islander people perceive wellbeing and quality of life (QOL) differently from the Western biomedical models of health underpinning existing QOL instruments. Any instrument to measure the wellbeing of Aboriginal and Torres Strait Islander people should be culturally appropriate and safe, include relevant dimensions, and be informed by their own values and preferences. Existing QOL instruments do not meet these standards. This study will generate a new preference-based wellbeing measure, WM2Adults, for Aboriginal and Torres Strait Islander adults, underpinned by their values and preferences.

**Methods:**

A mixed methods approach will be used; we will employ decolonising methodologies, privilege Aboriginal and Torres Strait Islander voices and perspectives, and adopt a strengths-based approach rather than a deficit lens. Yarning Circles will be conducted with Aboriginal and Torres Strait Islander people across Australia. A candidate item pool will be developed from these data, on which psychometric analysis and validity testing will be undertaken to develop a descriptive system. Following finalisation of the descriptive system, wellbeing states will be valued using a quantitative preference-based approach (best-worst scaling) with a diverse sample of Aboriginal and Torres Strait Islander adults (*n* = 1000). A multinomial (conditional) logit framework will be used to analyse responses and generate a scoring algorithm for the new preference-based WM2Adults measure.

**Discussion:**

The new wellbeing measure will have wide applicability in assessing the effectiveness and cost-effectiveness of new programs and services for Aboriginal and Torres Strait Islander people. Results will be disseminated through journals, conferences and policy forums, and will be shared with Aboriginal and Torres Strait Islander communities, organisations and research participants.

## Background

Understandings of health and wellbeing are culturally bound [1, 2]. Indigenous paradigms commonly embrace a holistic worldview of health that is multidimensional and incorporates the physical, cultural, spiritual, social and ecological wellbeing of the individual and the community. This concept also acknowledges a connectedness between these factors whilst also recognising the health and wellbeing of Indigenous people may be adversely affected through colonisation, historical and transgenerational trauma, racism and ongoing marginalisation [3]. Increasingly research has highlighted Aboriginal and Torres Strait Islander peoples (hereafter respectfully referred to as Indigenous Australians) prioritisation of family and relationships, and the importance of maintaining cultural obligations and connections as important to their wellbeing [4]. Preserving Indigenous languages and connectedness to Country, as well as other aspects of culture, values, and spirituality, have been identified as key contributors to wellbeing for Indigenous peoples [2, 5–10].

In Australia, the quality of life (QOL) and wellbeing of Indigenous people are poorly understood as existing measurement instruments are underpinned by Western biomedical models of health. Most QOL instruments used in Australia have limited applicability for Indigenous Australians, as relevant dimensions of wellbeing and QOL that are important to Indigenous people are often missing as they were not considered in the development of the tools.

Broadly, QOL can be considered a multidimensional construct that considers wellbeing and physical, psychological, social and emotional functioning [11]. The World Health Organization suggests the definition of QOL is also influenced by an individual’s culture and value systems; hence the need to explore and understand QOL concepts within the context that relates to the individual, inclusive of their community and culture [12]. Similarly, there is no single widely accepted definition of ‘wellbeing’; it is also multidimensional and described as a state of health, happiness and contentment along with security, including social aspects of life [5]. In many settings, the terms ‘QOL’ and ‘wellbeing’ are used interchangeably, and Indigenous notions of health are encompassed in the broader concept of wellbeing, therefore we will use the term ‘wellbeing’ henceforth.

Decision making agencies, such as the National Institute for Health and Care Excellence (NICE) in the UK, or the Pharmaceutical Benefits Advisory Committee (PBAC) or Medical Services Advisory Committee (MSAC) in Australia recommend or mandate the use of Quality Adjusted Life Years (QALYs) to express QOL outcomes. A QALY provides a multidimensional estimate of health outcome that includes survival and quality of life. To date, QALYs have been defined exclusively in terms of health status; other non-health aspects of quality of life are not included. Recently, researchers have suggested that QALYs should be defined more broadly than simply health status, with expanded focus to also consider wellbeing dimensions. For example, for older people, dimensions of QOL focussed on wellbeing (e.g. control, self-care, independence) were more important than physical symptoms [13]. The ‘extending the QALY’ (E-QALY) [14] project also takes a broader perspective on what dimensions are relevant; it will include aspects of life that patients, social care users and carers think are important to them and are impacted by their health condition, the care or treatment they receive or their caring role, including those not related to health.

In our systematic review [15] we found that the use of existing instruments in culturally and linguistically diverse respondents mostly involves language translation [16], with the implicit assumption that culture and context do not influence the applicability of instruments in different respondent groups [17]. However, simply translating Western measures into other contexts fails to capture critical concepts and dimensions relevant to other populations [18]. Aspects of life impacting on Indigenous Australians wellbeing [10] are simply not captured by existing biomedically focussed measures.

To be effective, measures of wellbeing for Indigenous people must privilege Indigenous voices to ensure they measure aspects of life valued by Indigenous people. As Kite et al. state “Privileging Indigenous peoples’ voices and gathering culturally specific expressions, understandings and knowledge of their circumstances may also assist in defining the factors that enhance or diminish Indigenous peoples QOL” [19]. Therefore, it is imperative that wellbeing concepts relate to, and incorporate, an individual’s community and culture and that these are captured in tools and instruments to measure wellbeing. Instruments should be valid, robust and include domains of wellbeing that are most relevant to Indigenous people [4]. It is vital that any measure not only captures an individual’s perceptions, but also conceptual notions of wellbeing in the context of their own culture and value systems [20]. To reflect this central notion of what is important for Aboriginal and Torres Strait Islander people in terms of their own wellbeing, we have called our study, and the resulting instrument “What Matters”.

### Study aims

The What Matters study will develop a new instrument to measure and value wellbeing dimensions that are important to Indigenous Australians with the end goal of improving the health and wellbeing of Indigenous Australians. To achieve this end goal, we need to improve the relevance and transparency of health decision making, and the new instrument will help facilitate this. Specifically, we will:

1. Identify the aspects of wellbeing that are important for Aboriginal and Torres Strait Islander people;
2. Develop and validate a descriptive system for a new wellbeing instrument that appropriately captures these aspects; and
3. Develop a preference-based scoring system for the new instrument that is underpinned by Aboriginal and Torres Strait Islander values and preferences, that can also be used in health system resource allocation frameworks.

## Methods/design

### Overview of approach and methods

**Fig. 1:**
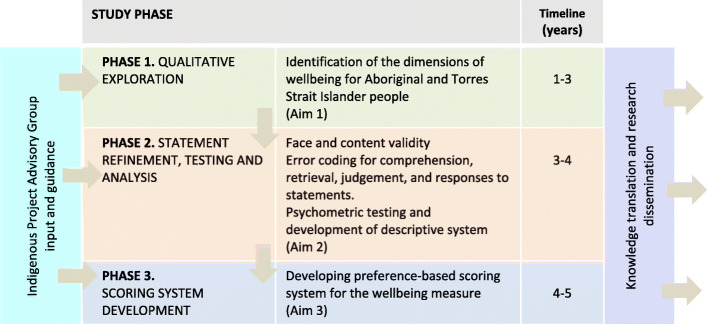
Overview of the three project phases

This is a 3-phase mixed methods project being conducted over 5 years (Fig. 1) which includes qualitative (Yarning Circles, semi-structured interviews), and quantitative psychometric and preference-based methods, including best-worst scaling (BWS) surveys.

The overall study methodology, research processes and outputs will be guided by the approaches and values of the Aboriginal and Torres Strait Islander communities they are intended to benefit.

Our approach is framed within Taylor’s construct of a ‘recognition space’ [21] – a shared conceptual space where the needs and values of governments and Indigenous people can converge, and commonly acceptable outcomes can be negotiated. This study will be conducted in accordance with the Values and Ethics of Aboriginal people as described in the National Health and Medical Research Council (NHMRC) Guideline for the Ethical Conduct in Aboriginal and Torres Strait Islander Health Research [22], and the Medical Journal of Australia (MJA) guidelines for conducting research among Indigenous people [23].

#### Decolonising methodologies

We have very deliberately adopted decolonising research methodologies in our What Matters study. Decolonizing research methodologies embrace approaches that prioritise Indigenous voices, and re-balance historical Western research practices. Central to this is Indigenous people being at the centre of research, to reclaim the space and control over the research that involves them [24]. This approach does not exclude non-Indigenous researchers, rather it repositions them as contributors to the research whilst ensuring Indigenous voices and perspectives are at the forefront of all aspects of the research process.

#### Indigenous project advisory group

As recommended [23] we have established an Indigenous Project Advisory Group (IPAG) to help shape our research methods and objectives, and to contribute to data interpretation. The IPAG, consisting of key Indigenous stakeholders and community members will provide congruency across the project regarding the development of the What Matters wellbeing measure. The IPAG is vital to the successful completion of the project, and will meet to provide advice twice a year, with out of session communication sought as needed.

We have also established an Indigenous Researchers Group comprising study investigators and research staff, who will provide additional day to day input and guidance on data collection, analysis, and interpretation.

### Phase 1: qualitative exploration of wellbeing (aim 1)

#### Research design

This phase will address Aim 1 and involve a large qualitative study that privileges the voices of Indigenous Australian adults about what matters in their lives and is important to, and impacts on, their wellbeing.

#### Data collection

We will recruit Indigenous adults from across Australia to participate in at least 30 Yarning Circles of around 8–10 people each. ‘Yarning’ methodology is a recognised culturally-appropriate style of communication and is used to gather information through sharing knowledge. This method of gathering information respects Indigenous Australians’ oral traditions, values, and privileges Indigenous Australian knowledge [25–27].

Purposive sampling will be used to ensure a maximally diverse range of age, gender, remoteness, and geographic distribution in the sample to ensure we capture a diverse range of views on aspects of wellbeing that are important for Indigenous Australians. The estimated sample size is based on our previous work, though the final number of groups and participants will depend on when data saturation is reached, defined as the point when little or no new outcomes or issues are emerging.

The Yarning Circle question guide will be based on our systematic reviews [10, 15] and previous studies [4, 7]; it will broadly cover aspects of wellbeing important for Indigenous Australians including but not limited to physical and psychological functioning, social and community roles, connection to land, family and spirituality, and any other aspects that arise during discussions. All Yarning Circles will be led by Indigenous researchers trained in qualitative research. Face to face semi-structured interviews will be conducted to supplement the data from the Yarning Circles. We will also collect sociodemographic information from participants.

#### Data analysis

Yarning Circles and semi-structured interviews will be audio recorded and transcribed. Transcripts will be imported into NVIVO12 software for analysis. We will extract wellbeing dimensions and use an adapted grounded theory approach and thematic analysis [28] to identify the reasons, values, and beliefs underpinning their choice of dimensions, an approach with which our team have extensive experience. Transcripts will be reviewed line-by-line to inductively identify concepts/themes, and be compared within and across respondent groups, to build a coding scheme. Through a process of constant comparisons, analytical themes will be developed. We will employ investigator triangulation and discuss with the Indigenous Project Advisory Group and the Indigenous Researchers Group to ensure findings capture the full breadth and depth of data. In line with Indigenous research principles, and a decolonising research approach, our analysis will be led by Aboriginal and Torres Strait Islander researchers and conducted collaboratively and iteratively to ensure that Indigenous voices and worldviews are privileged throughout the analysis process.

Following thematic analysis, we will develop a series of wellbeing statements generated from the Yarning Circles and individual interviews. These statements will be developed and refined in an iterative manner in partnership with the Indigenous Researchers Group and the Indigenous Project Advisory Group. This iterative process will develop and review draft, strengths-based wellbeing statements for potential inclusion in the WM2Adults wellbeing measure, and develop draft response scales for the statements (e.g. frequency (never, often, always), or severity (none, a little, a lot) based scales) to be evaluated in Phase 2.

### Phase 2: statement refinement, psychometric testing & descriptive system development (aim 2)

#### Research design

This Phase will evaluate the wellbeing statements from Phase 1 using Think Aloud methods and an online survey to assess the interpretation and understandability, as well as psychometric properties of the newly developed descriptive system.

#### Data collection

##### Think aloud study

A Think Aloud study will be conducted with Indigenous adults (*n* = 15) to assess the outcomes of Phase 1, for *face validity,* by whether statements were difficult to interpret and *content validity,* by whether respondents report missing dimensions. We will also assess the appropriateness of the statement response scales. Statement wording and response scales will be modified as needed based upon respondent feedback [29, 30].

##### Online survey

An online survey of *n* = 300 Aboriginal and Torres Strait Islander adults will be conducted where participants will complete all wellbeing statements using response scales finalised after the Think Aloud study and indicate the relevance of statements. Respondents will be recruited via online panels and through existing investigator networks.

#### Data analysis

##### Think aloud study

Thematic analysis will be conducted of the transcripts from the Think Aloud study. We will also use an existing Think Aloud error coding sheet, to assess if participants have any *comprehension, retrieval, judgement, response* or *struggle* issues with our statements.

##### Online survey

Usual conceptualisations of construct validity (e.g. convergence between instruments) may not be relevant, as we know existing instruments do not consider dimensions of wellbeing relevant for Aboriginal and Torres Strait Islander people. Instead, we will consider analyses at the item and dimension level to assess reliability and validity, including the extent of missing data (< 5%); item redundancy (inter item correlation < 0.75); endorsement frequencies and floor/ceiling effects (< 80%). We will also explore differences across sociodemographic groups.

Exploratory Factor Analysis (EFA) will initially be used to examine the dimensionality of the wellbeing statements. EFA tests the dimensionality of groups of items without imposing a pre-specified structure of dimensions. Items that do not load on any factor will be considered for exclusion from the measure, but other information will also be considered to ensure that the EFA does not exclude crucial items (e.g. identified in Phase 1). Confirmatory factor analysis (CFA) may then be used, if appropriate, to test the preferred factor structure. A range of statistics will be used to assess model fit and guide dimension development. We will also use of Item Response Theory (IRT) methods as a guide to wellbeing statement performance and selection, which will inform decision making about which statements are included in the final measure.

### Phase 3: scoring system development (aim 3)

#### Research design

Phase 3 will address Aim 3 and will lead to the development of a preference-based scoring system for the WM2Adults wellbeing measure, by estimating the relative weight assigned to each attribute and level defined by the descriptive system. We will employ Think Aloud methods to help determine the appropriate quantitative preference elicitation method and inform the design of the preference survey. We will use best practice methods for the design and analysis of Best Worst Scaling surveys (BWS) [31] and follow the approach taken in other similar projects [32–34]. This approach will allow development of a preference based scoring system to estimate overall, and dimension-specific wellbeing scores; and, potentially a quantitative estimation of relative health and wellbeing (analogous to, but broader than QALYs), if this is deemed appropriate by the research team and Indigenous Project Advisory Group.

#### Data collection

##### Think aloud study

A Think Aloud study will be conducted with Indigenous adults (*n* = 15) to help decide on the approach to be used for the quantitative preference elicitation survey. We will ask about ease of completion, understandability and preferred approach to inform a final decision about survey design [29, 30].

##### Preference survey

(Best Worst Scaling –Profile Case (Case 2- BWS) survey): We will recruit a minimum of 1000 adults, through panel providers and survey companies, with experience of working with Indigenous Australians, as well as through Investigator networks. Surveys will be conducted using both online and face to face modes of administration. A broad range of respondents will be included to enable us to examine any differences across sociodemographic and geographic groups.

#### Sample size

**Fig. 2:**
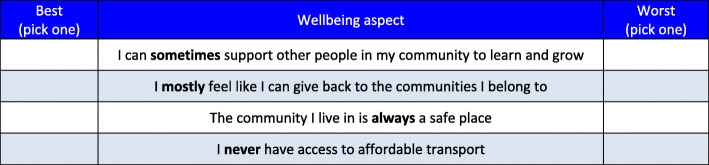
An example profile case (Case 2) BWS task

The optimal sample size for a BWS task is dependent upon the final number of dimensions and levels to be included in the wellbeing measure preference descriptive system, [35] as determined by Phase 2. It is not feasible to present all combinations of wellbeing statements to participants. We will use an efficient fractional factorial design [36]. For example, a fractional design of 150 scenarios (each with different combinations of wellbeing dimensions (e.g. Figure 2), can be blocked into 15 blocks of 10 scenarios and is sufficient to ensure an orthogonal main effects plan which maintains orthogonality and level balance [36]. Given large sample properties can be achieved with 50 respondents per block [31, 32], a design with 15 blocks and at least 65 respondents/block (*n* > 975) will be robust enough to estimate main effects, first order interactions and examine differences between participant subgroups. This sample size is also consistent with previous successful applications of this method [32, 34, 37].

#### Analysis

To ensure we maintain a decolonising approach and privilege Aboriginal and Torres Strait Islanders voices and perspectives, the methods, the analysis and results from the BWS as outlined below will be discussed throughout with the Indigenous Project Advisory Group and the Indigenous Researchers Group. The analysis and interpretation will be undertaken iteratively to ensure that Indigenous voices and worldviews are privileged throughout the analysis process.

We will apply a multinomial logit (conditional logit) modelling framework to analyse BWS responses collected from the survey of Indigenous adults. Random effect utility functions will be estimated following Random Utility Theory’s premise that the utility that an individual attaches to an attribute/level in a choice scenario is comprised of an explainable (fixed) component and an unexplainable (random) component.

Paired and marginal models for the prediction of wellbeing values will be estimated using data from the BWS questions. The BWS will first be analysed using conditional logistic regression models. These will be used to estimate paired (maxdiff) models where the best-worst pair is the unit of analysis, and sequential best-worst models where the dimension level is the unit of analysis [31, 32, 37]. Preference heterogeneity will be investigated via covariate adjusted regression, random parameter versions of these models and latent class analysis [37]. Values will be obtained for all possible states defined by the descriptive system using the marginal sequential or paired (maxdiff) model suggested by the BWS data.

The numerical estimates from the BWS task are initially anchored to the least valued dimension level. Since these estimates are on an interval scale, a linear transformation can be applied to the dimension level estimates to ensure that the highest wellbeing state (i.e. the sum of the best level values of all dimensions) takes the value 100 and the lowest wellbeing state takes the value 0. A similar process can be applied to each separate dimension, such that a dimension specific score on a 0–100 scale can also be estimated.

If, after discussion and consultation with our Indigenous Project Advisory Group and the Indigenous Researchers Group, it is decided that the measure should also be able to estimate outcomes akin to quality adjusted life years, additional transformations could be applied to the BWS data such that 0 represents the state ‘dead’, instead of the lowest wellbeing state from the measure. One method for achieving this involves rescaling the BWS estimates using the results obtained from a second preference task. This can been achieved by valuing a small number of wellbeing states, including the worst wellbeing state, from the new measure using a time trade off or a standard gamble exercise and then using those wellbeing state values to rescale the original BWS estimates to ensure that the 0 represents dead, as done in several previous studies [37–39].

The research team may consider conducting a second preference survey to allow this rescaling of the BWS estimates. If undertaken, it would include a small subsample of respondents who consent to participate in a follow-up study via interview, and the actual valuation approach will be refined and revised based on consultation with the Indigenous Advisory Group and other stakeholders. Previous studies have indicated these tasks are feasible [40], and would need a relatively small sample size of around 50 participants [37, 38]. If conducted, different analytical approaches used by previous studies could be compared in terms of overall model fit and mean absolute errors (MAE) to determine the optimal rescaling approach [37, 39].

#### Patient and public involvement

The purpose is of this study is to develop a wellbeing measure that is grounded in the preferences and values of Aboriginal and Torres Strait Islander people. Outcomes of this project (the new WM2Adults measure) are explicitly underpinned by the experiences, values and preferences of Indigenous Australians. To this end, we have established an Indigenous Project Advisory Group (IPAG) to help shape our research methods and objectives, and to contribute to data interpretation. The IPAG, consisting of key Indigenous stakeholders and community members will provide congruency across the project regarding the development of the What Matters wellbeing measure. The IPAG is vital to the successful completion of the project, and will meet to provide advice twice a year, with out of session communication sought as needed. Additionally, we have explicitly adopted decolonising research methodologies that prioritise Indigenous voices, and re-balance historical Western research practices. Indigenous people are positioned at the centre of the research, to reclaim the space and control over the research that involves them [24]. This approach ensures Indigenous voices and perspectives are at the forefront of all aspects of the research process.

## Discussion and conclusion

Measures of wellbeing for Indigenous people must privilege Indigenous voices to ensure they measure aspects of life valued by Indigenous people. The new WM2Adults wellbeing measure will measure and value wellbeing dimensions that are important to Aboriginal and Torres Strait Islander people with the preference-based scoring system that is underpinned by Aboriginal and Torres Strait Islander values and preferences. We will use a rigorous mixed methods approach, employing decolonising methodologies that prioritise Indigenous voices, values and preferences. Results will be disseminated through Indigenous health, quality of life research and health economics journals and through professional conferences and policy forums, and through Aboriginal and Torres Strait Islander communities, organisations and research participants. It will have wide applicability in assessing the effectiveness and cost-effectiveness of new programs and services for Aboriginal and Torres Strait Islander people and direct engagement with policy makers and key government agencies will also be undertaken. A website will be developed to facilitate access to the new wellbeing instrument and for publishing outcomes and key findings from the project for Aboriginal and Torres Strait Islander participants, their families and communities and the general public.

## Data Availability

Not applicable.
